# SETDB1 interactions with PELP1 contributes to breast cancer endocrine therapy resistance

**DOI:** 10.1186/s13058-022-01520-4

**Published:** 2022-04-08

**Authors:** Zexuan Liu, Junhao Liu, Behnam Ebrahimi, Uday P. Pratap, Yi He, Kristin A. Altwegg, Weiwei Tang, Xiaonan Li, Zhao Lai, Yidong Chen, Liangfang Shen, Gangadhara R. Sareddy, Suryavathi Viswanadhapalli, Rajeshwar R. Tekmal, Manjeet K. Rao, Ratna K. Vadlamudi

**Affiliations:** 1 Division of Reproductive Research, Department of Obstetrics and Gynecology, University of Texas Health San Antonio, 7703 Floyd Curl Drive, Mail Code 7836, San Antonio, TX 78229-3900 USA; 2 Department of Oncology, Xiangya Hospital, Central South University, Changsha, 410008 Hunan People’s Republic of China; 3 Department of Neurosurgery, Xiangya Hospital, Central South University, Changsha, 410008 Hunan People’s Republic of China; 4 Department of Obstetrics and Gynecology, Affiliated Hospital of Integrated Traditional Chinese and Western Medicine, Nanjing University of Chinese Medicine, Nanjing, 210028 People’s Republic of China; 5 Greehey Children’s Cancer Research Institute, University of Texas Health San Antonio, San Antonio, TX 78229 USA; 6 Mays Cancer Center, University of Texas Health San Antonio, San Antonio, TX 78229 USA; 7 Audie L. Murphy Division, South Texas Veterans Health Care System, San Antonio, TX 78229 USA; 8 Dept of Population Health Sciences, University of Texas Health San Antonio, San Antonio, TX 78229 USA

**Keywords:** SETDB1, Akt, PELP1, Breast cancer, Therapy resistance

## Abstract

**Background:**

Methyltransferase SETDB1 is highly expressed in breast cancer (BC), however, the mechanisms by which SETDB1 promotes BC progression to endocrine therapy resistance remains elusive. In this study, we examined the mechanisms by which SETDB1 contribute to BC endocrine therapy resistance.

**Methods:**

We utilized therapy sensitive (MCF7 and ZR75), therapy resistant (MCF7-TamR, MCF7-FR, MCF7-PELP1cyto, MCF7-SETDB1) estrogen receptor alpha positive (ER^+^)BC models and conducted in vitro cell viability, colony formation, 3-dimensional cell growth assays to investigate the role of SETDB1 in endocrine resistance. RNA-seq of parental and SETDB1 knock down ER^+^ BC cells was used to identify unique pathways. SETDB1 interaction with PELP1 was identified by yeast-two hybrid screen and confirmed by immunoprecipitation and GST-pull down assays. Mechanistic studies were conducted using Western blotting, reporter gene assays, RT-qPCR, and in vitro methylation assays. Xenograft assays were used to establish the role of PELP1 in SETDB1 mediated BC progression.

**Results:**

RNA-seq analyses showed that SETDB1 regulates expression of a subset of estrogen receptor (ER) and Akt target genes that contribute to endocrine therapy resistance. Importantly, using yeast-two hybrid screen, we identified ER coregulator PELP1 as a novel interacting protein of SETDB1. Biochemical analyses confirmed SETDB1 and PELP1 interactions in multiple BC cells. Mechanistic studies confirmed that PELP1 is necessary for SETDB1 mediated Akt methylation and phosphorylation. Further, SETDB1 overexpression promotes tamoxifen resistance in BC cells, and PELP1 knockdown abolished these effects. Using xenograft model, we provided genetic evidence that PELP1 is essential for SETDB1 mediated BC progression in vivo. Analyses of TCGA datasets revealed SETDB1 expression is positively correlated with PELP1 expression in ER^+^ BC patients.

**Conclusions:**

This study suggests that the PELP1/SETDB1 axis play an important role in aberrant Akt activation and serves as a novel target for treating endocrine therapy resistance in breast cancer.

**Supplementary Information:**

The online version contains supplementary material available at 10.1186/s13058-022-01520-4.

## Background

Breast cancer (BC) is the most common malignancy in women, and globally accounts for approximately 685,000 deaths annually. The majority of BC (70%) is estrogen receptor alpha positive (ER^+^) upon diagnosis. The current standard of care includes the use of endocrine therapies such as tamoxifen the oldest and most prescribed selective estrogen receptor modulator, anti-estrogens, selective estrogen receptor degraders, and aromatase inhibitors which are widely used for the treatment of ER^+^ BC. However, over time approximately 30–40% of patients develop resistance to these treatment options, and this represents a major clinical problem [1–3]. Several lines of evidence suggest that multiple pathways including oncogenic growth factor signaling [4, 5], epigenetic changes [6], activation of the PI3K/Akt/mTOR pathway [7], and alterations of levels of ER coregulators [8] contribute to endocrine therapy resistance. However, the molecular mechanisms that contribute resistance to endocrine therapy are not completely understood.

SETDB1 (SET domain bifurcated 1), is a methyltransferase involved in di and tri-methylation of H3K9 and is implicated in repression of transcription [9]. Deregulation of SETDB1 expression occurs in many cancers [10]. In fact, SETDB1 is amplified in triple negative breast cancers (TNBC) [11] and is shown to regulate TNBC metastasis by enhancing stem-cell-like properties and modulating the epithelial-mesenchymal transition (EMT) program [12]. Recent studies also suggest that SETDB1 is localized both in the nucleus and cytoplasm. Further, SETDB1 mediated Akt K64 methylation promotes Akt hyperactivation resulting in cancer progression [13]. However, the molecular mechanisms by which SETDB1 contributes to ER^+^ BC progression to endocrine therapy resistance is poorly understood.

Proline-, glutamic acid-, and leucine-rich protein 1 (PELP1) is a scaffolding protein that functions as a coregulator of several nuclear receptors including ER [14]. PELP1 oncogenic signaling is implicated in BC progression [15, 16] and transgenic mice with PELP1 overexpression in the mammary glands develop mammary gland carcinoma [17]. PELP1 is a known prognostic indicator of poorer BC survival [18], and its dysregulation has been shown to contribute to BC therapy resistance [19, 20]. Furthermore, patients whose breast tumors exhibit high levels of cytoplasmic PELP1 respond poorly to tamoxifen [21]. However, the mechanisms by which PELP1 contributes to endocrine therapy resistance remains elusive.

In this study, we found that PELP1 functions as an important regulator of SETDB1. RNA-seq studies revealed that SETDB1 participates in the regulation of a subset of ER target genes that contribute to tamoxifen therapy resistance. Mechanistic studies showed that PELP1 interacts with SETDB1 and plays a critical role in the activation of Akt by facilitating Akt methylation. Further, SETDB1 overexpression promoted tamoxifen therapy resistance and PELP1 is necessary for SETDB1 mediated Akt signaling leading to therapy resistance in ER^+^ BC cells. Our studies have identified a novel mechanism by which the PELP1/SETDB1 axis contributes to tamoxifen therapy resistance via activation of Akt. These findings are functionally significant as increased expression of both PELP1 and SETDB1 occur in ER^+^ BC. Therefore, the PELP1-SETDB1 axis could serve as a potential therapeutic target for treating endocrine therapy resistance.

## Methods

### Cell cultures and reagents

MCF7, ZR75, and HEK293T cell lines were purchased from the American-Type Culture Collection (ATCC) and maintained in ATCC recommended medium. All model cells utilized were free of mycoplasma contamination and STR DNA profiling of the cells was used to confirm identity. MCF7-TamR cells were cultured in medium supplemented with 1 µmol/L of tamoxifen (H7904, Sigma, St. Louis, MO). MCF7-FR cells were cultured in medium supplemented with 1 µmol/L of fulvestrant (MedChem Express, Monmouth Junction, NJ) for 6 months to develop resistance. The SETDB1 antibody (11231-1-AP) was obtained from Proteintech (Rosemont, IL). The PELP1 antibody (A300-180A) was purchased from Bethyl Laboratories (Montgomery, TX). The ERα (04-820) and β-Actin antibodies (A-2066) were purchased from Millipore Sigma (Burlington, MA). The phospho-ER (Ser167) antibody (PA5-37570) was purchased from Invitrogen (Waltham, MA). The mCherry antibody (632543) was obtained from Takara Bio (San Jose, CA). The GFP antibody (632460) was obtained from Clontech. Antibodies for GST (2624), p-Akt (4060), Akt (9272), tri-methyl lysine motif (K-me3,14680) and GAPDH (8884) were purchased from Cell Signaling Technology (Beverly, MA). For IHC analysis, Ki67 antibody (ab16667) was purchased from Abcam (Cambridge, MA) and p-Akt (05-1003) antibody was purchased from Millipore Sigma.

### Generation of model cell lines

MCF7 and ZR75 cells stably expressing SETDB1-shRNA were generated using lentivirus expressing SETDB1 shRNA (TRCN0000276169, TRCN0000276105, Sigma). MCF7 and ZR75 cells stably expressing PELP1-shRNA were created using validated human specific lentiviral PELP1-shRNA particles (TRCN0000159883, Sigma). MCF7 cells expressing T7-cyto-PELP1 were earlier described [22]. MCF7 and ZR75 cells stably expressing SETDB1 were generated using pLV-Bsd-EF1A-SETDB1 vector. HEK293T cells expressing GFP-SETDB1 were generated using pLV-Bsd-EF1A-EGFP-SETDB1 vector. MCF7, ZR75 and HEK293T cells expressing mCherry-cyto-PELP1 were generated using pLV-Hygro-EF1A-Cherry-PELP1-Cyto-NLSmt (KKK to EEE) vector. HEK293T cells stably expressing GST-Akt were created using the lentiviral vector pLV-Hygro-EF1A-GST-AKT. Lentiviral particles expressing non-targeted shRNA (SHC016-1EA, Sigma), GST-vector (pLV-Puro-EF1A-GST vector), mCherry-vector (pLV-mCherry-Puro-EF1A vector) were used to generate control cells. All the vectors are custom made by Vector Builder (https://en.vectorbuilder.com). Stable clones were generated using puromycin (1 μg/mL) or hygromycin (100 μg/mL) selection and pooled clones were used for all studies.

### Reporter gene assays

HEK293T cells stably expressing ERE firefly luciferase reporter and ERα were transfected with SETDB1 expressing vector or control vector using Turbofect transfection reagent (Thermo Fisher Scientific, Waltham, MA). MCF7 cells stably expressing SETDB1-shRNA or non-targeted shRNA were transduced with ERE luciferase reporter. The Renilla reporter plasmid was co-transfected and used for data normalization. After 48 h, cells were treated with E2 (10^–8^ M) for 24 h and luciferase activity was measured using the Dual luciferase assay system (Promega, Madison, WI) using a luminometer.

### Yeast-two hybrid, immunoprecipitation and Western blot assays

Mapping of the PELP1-SETDB1 interaction region using yeast-two hybrid assay was done as described [23]. Yeast cells were co-transfected with a gal4 activation domain (GAD) fusion GAD-SETDB1, along with gal4 binding domain (GBD) vector or GBD fusions of various domains of PELP1. Growth was recorded after 72 h on selection plates lacking leucine and tryptophan (− LT) or adenine, histidine, leucine, and tryptophan (− AHLT). BC cells were stimulated either with E2 or 10% fetal bovine serum (FBS). For E2 stimulation, MCF7 and ZR75 cells were cultured in Dextran Coated Charcoal stripped serum (DCC) supplemented medium for 48 h and then stimulated with E2 (10^–8^ M). To simulate the growth factor driven signaling, MCF7 and ZR75 model cells were serum-starved for 24 h and stimulated with medium containing 10% serum. Cell lysates were prepared by RIPA buffer (Thermo Fisher Scientific) containing protease and phosphatase inhibitors and Western blot analysis was performed. For co-immunoprecipitation analysis, the lysates were incubated with indicated antibody or IgG control overnight at 4 °C, and then incubated with Protein A/G beads (Thermo Fisher Scientific) for 2 h, overnight incubation with GST beads (17-0756-01, GE Healthcare, Chicago, IL) or T7 Tag Antibody Agarose (69026, Novagen) or GFP-TRAP and RFP-TRAP Agarose (ChromoTek) at 4 °C. Interactions were analyzed by Western blotting using indicated antibodies. For GST pull-down assays, GST-tagged Akt1 protein was purified from HEK293T cells.

### In vitro methylation assay

Purification of PELP1 protein was done using baculovirus system as earlier described [24]. GST-tagged Akt1 protein was purified from HEK293T cells using GST-pull down assay. Recombinant SETDB1 protein was purchased from Active Motif (cat#31452, Carlsbad CA) and manufacturer’s protocol was followed for the methylation assay. Briefly, SETDB1, Akt and PELP1 proteins were incubated in 50 mM Tris–HCl (pH 8.6), 0.02% Triton X-100, 2 mM MgCl_2_, 1 mM TCEP, 50 µM S-adenosyl-methionine (SAM) buffer for 4 h at 37 °C. The reaction was stopped by the addition of Laemmli reducing sample buffer and run on 8% electrophoresis gel.

### Cell viability, clonogenic assays and three-dimensional cell culture

Cell viability was determined using MTT assay as described [25]. For the clonogenic assays, model cells (500 cells/well) were seeded in 6 well plates and survival was analyzed after 14 days. To test the drug effect on colony formation, the model cells were treated with E2 (10^–8^ M) in the presence or absence of tamoxifen or Mithramycin A (Millpore Sigma) for 5 days and allowed to grow for another 7 days. The cells were fixed in ice-cold methanol and stained with 0.5% crystal violet solution. For 3D cell culture, model cells suspended in matrigel (6 × 10^3^/40 μL) were plated onto a culture plate, incubated at 37 °C for 10 min to solidify and subsequently cultured for 10 days in DMEM/F12 growth medium. The relative size of the colonies was analyzed by ImageJ software.

### RNA-seq, bioinformatic and RT-qPCR analysis

Total RNA from MCF7 cells stably expressing control non-targeted shRNA or SETDB1 shRNA was isolated using RNeasy mini kit (Qiagen). The RNA-Seq library was prepared using Illumina TruSeq stranded mRNA Sample preparation kit (Illumina) and sequencing was performed at Greehey Children’s Cancer Research Institute Genome Sequencing Facility (UT Health, SA) using 50 bp single read sequencing module with Illumina HiSeq 3000 sequencing platform. Sequence reads were mapped to UCSC hg19 genome using TopHat2 aligner and quantified to NCBI RefSeq genes using HTSeq [26]. Differential expression analysis was conducted using DEseq2 [27] and significant genes with fold change > 2 and adjusted *p* value < 0.05 were used for interpreting functional enrichment pathways. Gene set enrichment analysis (GSEA) (http://www.broadinstitute.org/gsea/index.jsp) [28] was used to perform gene set enrichment analysis. The Pheatmap package (1.0.12) was used to create heatmaps of differential genes. PELP1 RNA-seq analysis results used in this study was previously published [29]. RNA-seq data has been deposited in the GEO database under GEO accession number GSE187398. Biological network integration for prediction of gene interaction was done by GeneMANIA prediction server [30]. Biomolecular interaction networks were visualized using Cytoscape (V.3.8.2) [31]. Tumor subgroup SETDB1 gene expression analyses was done using UALCAN [32]. Comparison of SETDB1 gene expression in normal and breast tumor tissues was done by TNMplot [33]. Gene-expression correlation analyses of TCGA breast cancer dataset was done using bc-GenExMiner 3.0[34]. For Real-time quantitative PCR (RT-qPCR) analyses total RNA was isolated using Trizol Reagent (Invitrogen). Reverse transcription was performed using SuperScript III First Strand kit (Invitrogen). RT-qPCR was done using SYBR Green (Thermo Fisher Scientific) with the primers included in the Additional file 1.

### In vivo xenograft studies

All animal studies were conducted after obtaining UT Health San Antonio IACUC approval, and in accordance with IACUC guidelines. SCID mice (*n* = 5, per group) were implanted with E2 pellet (cat# SE-121, Innovative Research of America, Sarasota, FL) as described previously [35]. Model cells MCF7-Control, MCF7-SETDB1, MCF7-PELP1-KD, MCF7-PELP1-KD + SETDB1 (2 × 10^6^ cells) were mixed with equal volume of matrigel and injected orthotopically into the mammary fat pads of 8-week-old SCID mice. Tumor growth was measured using calipers at weekly intervals, and tumor volume was calculated using a modified ellipsoidal formula: tumor volume = 1/2(L × W^2^), where W is the transverse diameter and L represents longitudinal diameter. At the end of the experiment, mice were euthanized, and tumors were processed for histological studies.

### Immunohistochemistry (IHC) analyses

Immunohistochemical analysis was conducted using an established protocol [25]. Briefly, sections were blocked with normal horse serum (Vector Labs, Burlingame, CA) followed by overnight incubation with primary antibody [Ki-67 (1:100); p-Akt(S473) (1:100)] and subsequent secondary antibody incubation for 30 min at room temperature. Percentage of Ki-67 positive cells and the staining intensity of p-Akt (S473) was calculated in five randomly selected microscopic fields. Quantification of D-HSCORE value was done by ImageJ software as described previously [36].

### Statistical analysis

The student’s *t*-test and one-way ANOVA were used to analyze the statistical differences with GraphPad Prism 7 software (GraphPad Prism Software, San Diego, CA). All the data represented in bar graphs are shown as mean ± SEM. A value of *p* < 0.05 was considered as statistically significant.

## Results

### SETDB1 knockdown reduces the growth of ER^+^ BC cells and decreases expression of ER target genes

**Fig. 1:**
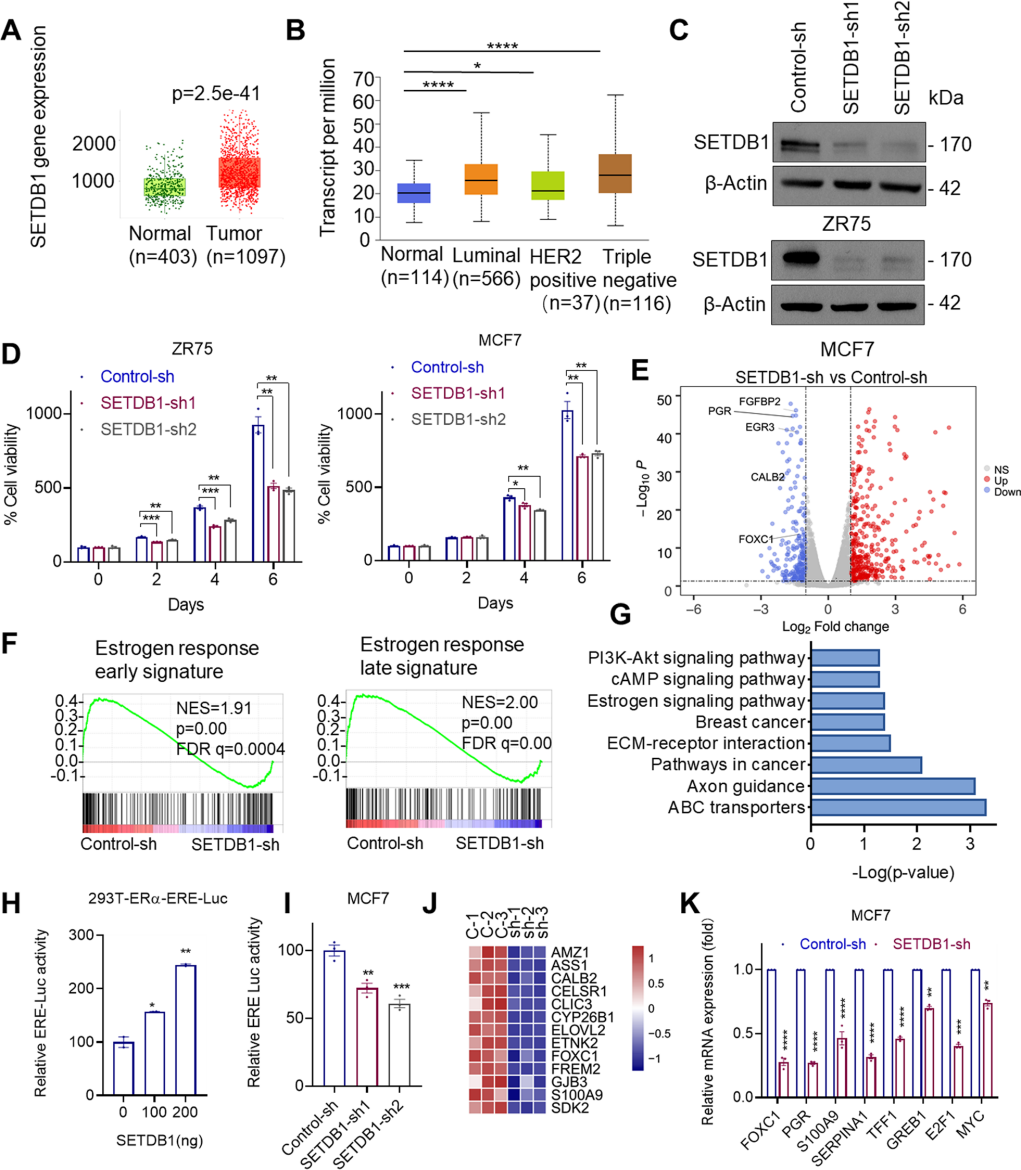
SETDB1 regulates ER driven transcription. **A**, **B** Expression level of SETDB1 between normal and BC tumor (**A**), and BC subtypes (**B**) were examined using online platforms TNM plot (**A**) and UALCAN portal (**B**). **C** Validation of SETDB1-KD by Western blotting using two independent shRNAs targeting SETDB1 in ZR75 and MCF7 cells. **D** Effect of SETDB1 KD on the cell viability of ER^+^ BC cells cultured in E2 (10^−8^ M) was measured by MTT assay. **E** MCF7 control-sh and SETDB1-sh cells were stimulated with E2 (10^−8^ M) for 8 h and then subjected to RNA-seq. Volcano plot of differentially expressed genes from RNA-seq data of MCF7 control-sh and SETDB1-sh cells. The x axis shows the log2 fold change and the y-axis shows the − log10 (*p*.value). The red dots represent significantly upregulated genes whereas the blue dots represent significantly downregulated genes upon SETDB1-KD. **F** GSEA enrichment plots of estrogen response signaling genes altered with SETDB1-KD. NES, normalized enrichment score. *p* values and FDR *q*.values were calculated using the GSEA package. **G** Representative KEGG pathways enriched (*p*.value < 0.05) in genes down-regulated upon SETDB1-KD. **H** 293 T-ERα-ERE-Luc cells were co-transfected with SETDB1 expressing vector or control vector along with pRL vector. After 48 h cells were stimulated with E2 for 24 h and luciferase activity was determined using Renilla dual luciferase assay system. **I** MCF7 cells that stably express SETDB1-shRNA were transduced with ERE-Luc, stimulated with E2 for 24 h and the reporter activity was determined. **J** Heat map depicting the expression levels of ER target genes from RNA-seq. **K** Selective ER target genes were validated by RT-qPCR in MCF7 control-sh and SETDB1-sh cells. Data was represented as mean ± SEM. *p < 0.05; **p < 0.01; ***p < 0.001; ****p < 0.0001

To study the status of SETDB1 expression in BC, we examined the degree of SETDB1 alterations using TNM-plot that enables comparison of gene expression between tumor and normal tissues [37]. Results showed that SETDB1 is highly expressed in BC compared to normal tissues (Fig. 1A). Further, analyses using TCGA data bases showed that SETDB1 is highly expressed in ER^+^ and triple negative breast cancer (TNBC) compared to normal breast (Fig. 1B). To understand the role of SETDB1 in ER^+^ BC, we have established ER + BC models with stable knockdown (KD) of SETDB1 by using two distinct validated SETDB1 shRNAs. Western blot analyses confirmed knockdown of SETDB1 in both MCF7 and ZR75 cells (Fig. 1C). When cultured in E2 supplemented medium, SETDB1 KD cells showed decreased growth rates compared to control cells (Fig. 1D).

To determine the mechanisms by which SETDB1 regulate the cell viability of ER^+^ BC cells, we performed RNA-Seq of MCF7 control and SETDB1-KD cells cultured in E2 supplemented medium. These studies identified 245 down-regulated genes and 409 upregulated genes upon SETDB1 KD (Fig. 1E). Gene set enrichment analysis (GSEA) revealed the positive correlation between SETDB1-regulated genes with signatures of early and late estrogen response targets (Fig. 1F). Further analysis using the KEGG pathway database confirmed that down-regulated genes upon SETDB1-KD were involved in ER-signaling, breast cancer, and PI3K-Akt signaling. (Fig. 1G). We then examined whether SETDB1 regulates ER transcription using ERE reporter assays. In HEK293T cells, expression of SETDB1 enhanced ER-driven ERE-Luc reporter activity in a dose dependent manner (Fig. 1H). Further, SETDB1-KD significantly reduced the ERE reporter activity in MCF7 cells (Fig. 1I). Moreover, analyses of the RNA-seq data confirmed down regulation of several known ER target genes (Fig. 1J). We also independently validated that SETDB1-KD reduces ER target genes (Fig. 1K). Collectively, these results suggest that SETDB1 regulates the expression of subsets of genes involved in ER^+^ BC progression.

### SETDB1 modulates tamoxifen response via Akt activation

**Fig. 2:**
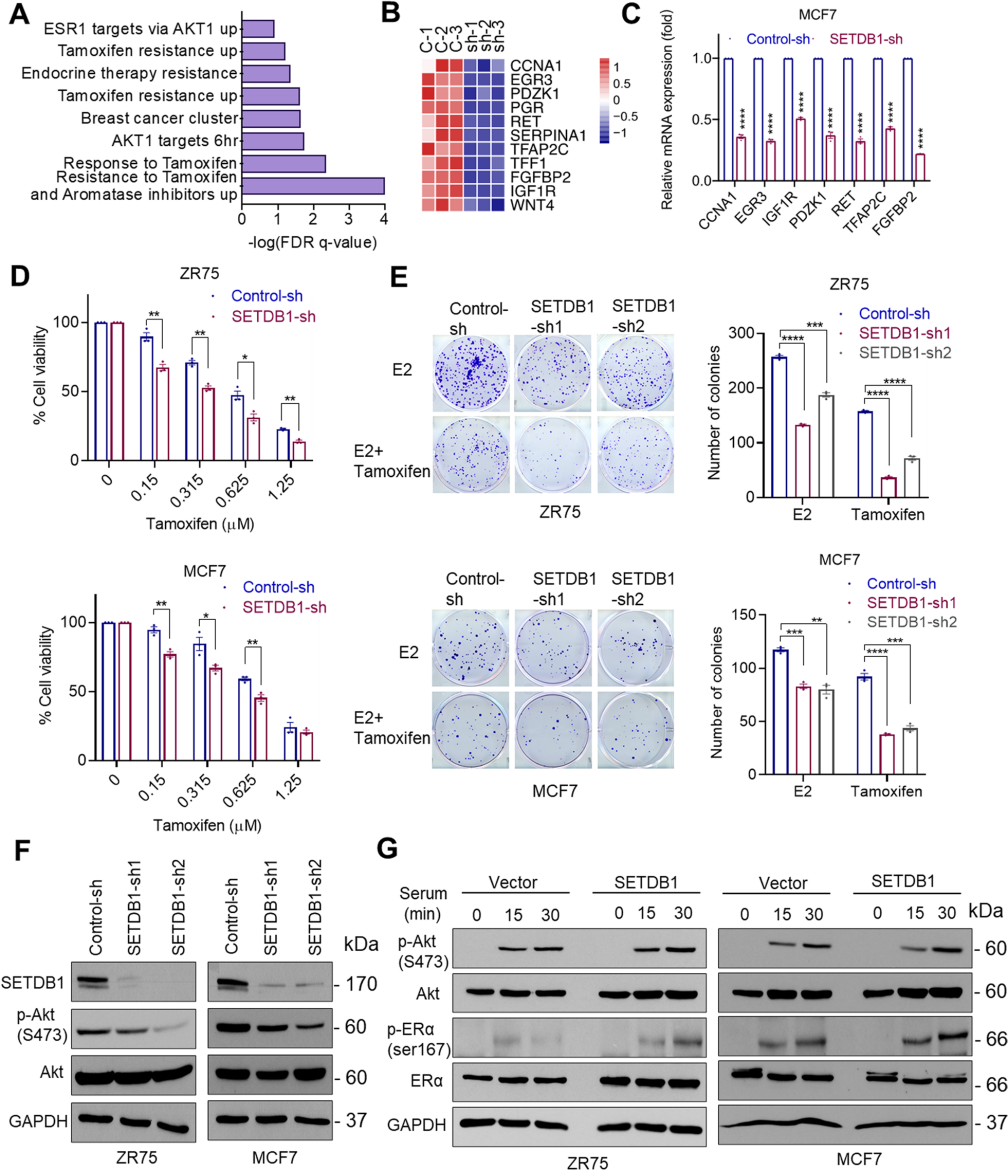
SETDB1 modulates tamoxifen response via Akt activation. **A** Gene set enrichment analysis of Akt signaling and tamoxifen resistance gene signatures upon SETDB1 KD were analyzed using RNA-seq data. **B**, **C** Effect of SETDB1 KD on selective tamoxifen resistance genes shown by heatmap (**B**) were validated using RT-qPCR analysis (**C**). **D** Effect of tamoxifen on the cell viability of control-sh and SETDB1-sh ZR75 and MCF7 cells was determined using the MTT assay. **E** Effect of tamoxifen on colony formation of control-sh and SETDB1-sh cells. Model cells were cultured in 5% DCC medium for 48 h, then treated with E2 (10^−8^ M) or Tamoxifen (2.5 μM). Shown are the results from one representative experiment of three replicates. **F** ZR75 and MCF7 cells stably expressing control-sh or SETDB1-sh were serum starved for 24 h and stimulated with 10% serum for 15 min. Effect of SETDB1-KD on Akt phosphorylation was analyzed by Western blotting. **G** ZR75 and MCF7 cells stably expressing control vector or SETDB1 vector were serum starved for 24 h and stimulated with 10% serum for 0, 15, and 30 min. Effect of SETDB1 overexpression on Akt downstream signaling was determined by Western blotting. Data was represented as mean ± SEM. *p < 0.05; **p < 0.01; ***p < 0.001; ****p < 0.0001

We next analyzed the SETDB1 regulated genes identified from RNA-seq analysis and found that these genes are positively correlated with the signatures of AKT pathway and tamoxifen resistance (Fig. 2A). Several genes known to be associated with tamoxifen therapy resistance were down regulated in SETDB1-KD cells (Fig. 2B). Further, we independently confirmed SETDB1 regulation of tamoxifen resistance genes by RT-qPCR (Fig. 2C). We then examined whether SETDB1 plays a role in tamoxifen response using cell viability and survival assays. Knock down of SETDB1 significantly enhanced the ability of tamoxifen to reduce cell viability (Fig. 2D) and cell survival compared to control cells (Fig. 2E). We next confirmed whether SETDB1 regulates activation of Akt1 in ER^+^ BC cells. Knockdown of SETDB1 in MCF7 and ZR75 cells reduced the levels of phospho-Akt compared to controls (Fig. 2F). Accordingly, overexpression of SETDB1 increased activation of Akt and phosphorylation of its substrate ER at Ser167 (Fig. 2G). Collectively, these results suggest that SETDB1 may participate in tamoxifen resistance by regulating Akt-mediated ER signaling.

### SETDB1 knockdown sensitizes therapy resistant cells to endocrine treatment

**Fig. 3:**
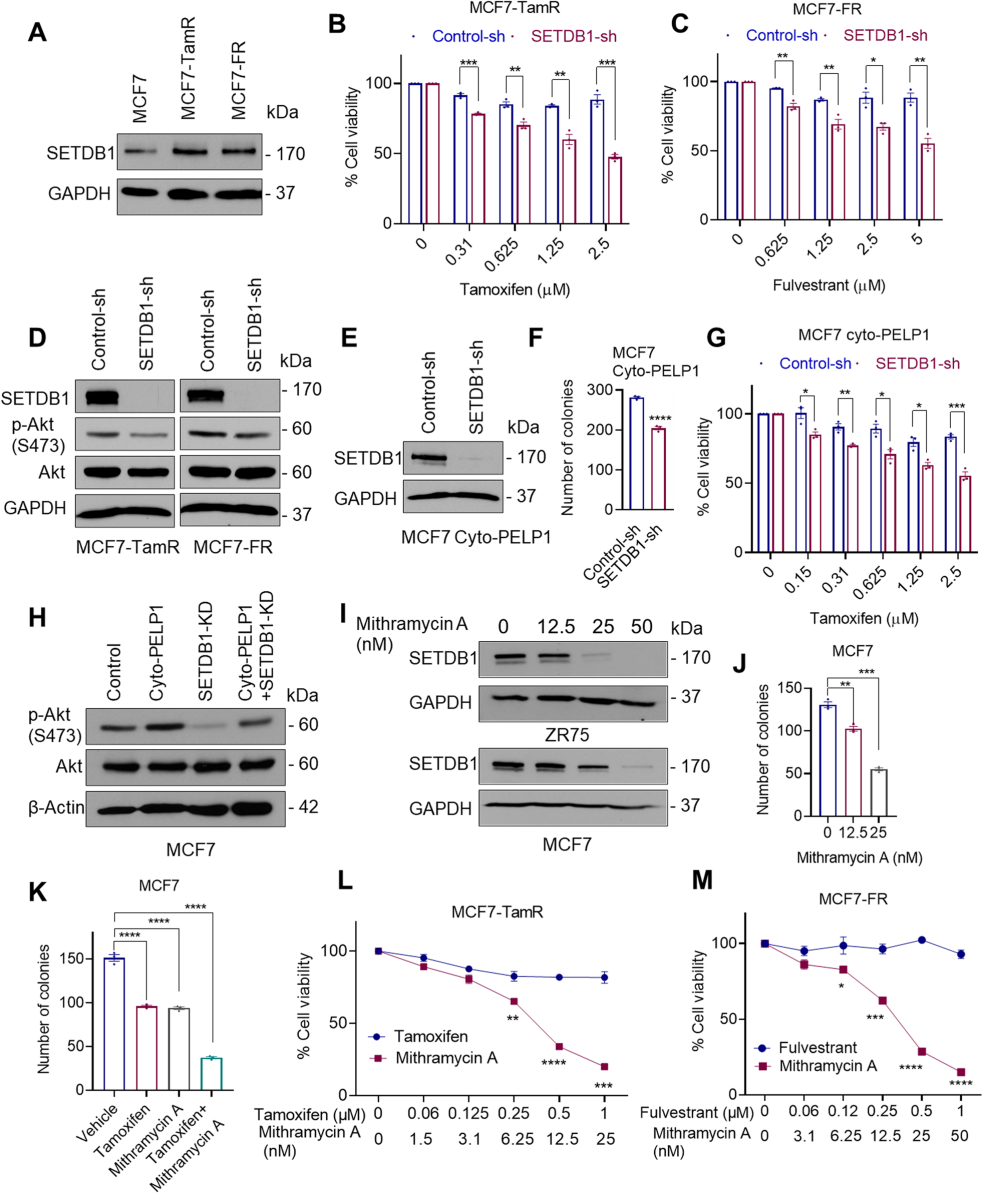
SETDB1 knockdown sensitizes therapy resistant cells to endocrine treatment. **A** SETDB1 expression in MCF7 and tamoxifen-resistant (TamR) or fulvestrant -resistant (FR) cells were examined by Western blotting. **B**, **C** Effect of tamoxifen or fulvestrant on the cell viability of MCF7-TamR (**B**) or MCF7-FR **(C**) that stably express control-sh and SETDB1-sh was determined using MTT assays. **D** SETDB1 expression was knocked down in MCF7-TamR and MCF7-FR cells and the status of Akt activation was measured using Western blotting. Cells were serum-starved for 24 h and stimulated with 10% serum for 15 min before being harvested and subjected to western blotting. **E** Confirmation of SETDB1-KD in MCF7-PELP1cyto cells. **F** Effect of SETDB1-KD on the colony formation of MCF7-PELP1cyto cells. **G** Effect of tamoxifen on the viability of PELP1cyto control-sh or SETDB1-sh cells was determined using MTT assays. **H** MCF7-PELP1cyto cells with/without SETDB1 KD were serum-starved for 24 h, stimulated with serum and total lysates were analyzed for Akt activation using Western blotting. **I** Cells were treated with indicated dose of Mithramycin A for 3 days, and the status of SETDB1 was determined using Western blotting. **J**, **K** Effect of Mithramycin A alone (**J**) or in combination with tamoxifen (**K**) on the cell survival was measured using colony formation assays. **L**, **M** Effect of Mithramycin A on the cell viability of MCF7-TamR (**L**) and MCF7-FR (**M**) cells was determined using MTT assays. Data was represented as mean ± SEM. *p < 0.05; **p < 0.01; ***p < 0.001; ****p < 0.0001

Since Akt1 activation is implicated in endocrine therapy resistance [38] and our RNA-seq data suggested that SETDB1 regulates Akt-mediated ER signaling; we examined the status of SETDB1 in endocrine therapy resistant model cell lines. Western blot analysis confirmed tamoxifen-resistant and fulvestrant-resistant MCF7 models have higher levels of SETDB1 compared to parental MCF7 cells (Fig. 3A). Cell viability assays showed that knockdown of SETDB1 in MCF7-TamR and MCF7-FR cells sensitized them to tamoxifen and fulvestrant treatment respectively (Fig. 3B, C). Further analyses using Western blotting showed decreased Akt1 activation in SETDB1-KD cells compared to controls (Fig. 3D). Further, previous studies have shown cytoplasmic localization of PELP1 occurs in breast tumors and contributes to endocrine resistance via Akt1 activation [22]. Therefore, we next examined if SETDB1 plays a role in the activation of Akt1 using PELP1 cytoplasmic localization (PELP1cyto) model cells. We observed that SETDB1-KD substantially decreased the colony formation ability of MCF7-PELP1cyto cells (Fig. 3E, F) and sensitized them to tamoxifen treatment compared to control cells (Fig. 3G). Further, knockdown of SETDB1 substantially reduced Akt phosphorylation mediated by PELP1 cytoplasmic localization (Fig. 3H). We confirmed the role of SETDB1 in endocrine therapy resistance using Mithramycin A, a small molecular inhibitor previously shown to reduce SETDB1 expression via transcriptional down regulation [39]. In ER^+^ BC MCF7 and ZR75 cell lines, treatment with Mithramycin A significantly reduced SETDB1 expression (Fig. 3I). Further, Mithramycin A treatment significantly reduced the colony formation of BC cells in a dose dependent manner (Fig. 3J) and enhanced the efficacy of tamoxifen (Fig. 3K). Mithramycin A treatment reduced cell viability of MCF7-TamR and MCF7-FR cells when compared to tamoxifen or fulvestrant treatment respectively (Fig. 3L, M). Collectively, these data suggest that SETDB1 mediated activation of Akt1 may contributes to endocrine therapy resistance.

### PELP1 is a novel interacting protein of SETDB1

**Fig. 4:**
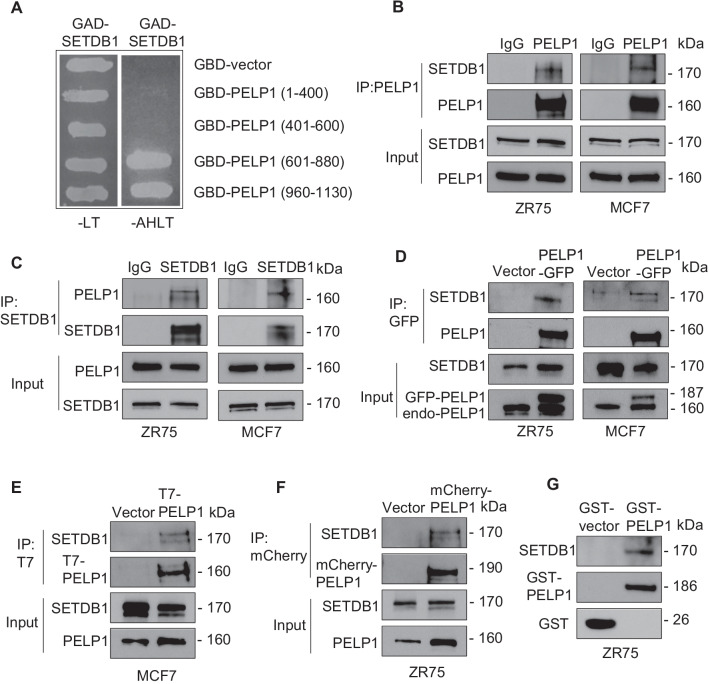
PELP1 is a novel interacting protein of SETDB1. **A** Mapping of PELP1-SETDB1 interaction region using yeast two hybrid assay. GBD fusions of PELP1 fragments were used to determine the PELP1 binding region in SETDB1. Positive interactors were selected on agar plates lacking either leucine and tryptophan (-LT) or adenine, histidine, leucine, and tryptophan (-AHLT). **B**, **C** Total lysates from ZR75 and MCF7 cells cultured in 10% serum were subjected to immunoprecipitation using PELP1 (**B**) or SETDB1 **(C**) antibody. IgG antibody was used as a negative control. SETDB1 and PELP1 interaction was confirmed by Western blotting. **D** ZR75 and MCF7 cells cultured in 10% serum were transfected with vector or GFP-tagged PELP1. The interaction of GFP-tagged PELP1 with SETDB1 was analyzed by immunoprecipitation using GFP-TRAP beads. **E**, **F** Total cell lysates from MCF7 cells transfected with T7-PELP1cyto or ZR75 cells transfected with mCherry-PELP1cyto were cultured in 5% DCC medium for 48 h, stimulated with E2 for 15 min, and then subjected to immunoprecipitation using epitope tag antibodies. Interaction between cytoplasmic PELP1 and SETDB1 was analyzed by Western blotting. **G** Total lysates from ZR75 cells cultured in 10% serum were subjected to GST pull-down assays using the purified GST vector or GST-PELP1 full length proteins. SETDB1 and PELP1 binding was analyzed by Western blotting

Our ongoing yeast two-hybrid library screening studies identified SETDB1 as a potential PELP1‐binding protein. To further confirm the specificity and domains of PELP1 interactions with SETDB1, we repeated the yeast-two-hybrid screen assay with co-transformation of a GAD-SETDB1 plasmid along with vectors expressing various domains of PELP1 as GBD fusions. We monitored the interactions via survival assays in selection medium using yeast cells which stably express adenine and  histidine, nutrient reporter genes under the control of GAL response elements. The GBD-PELP1-601-880 or GBD-PELP1-960-1130 and GAD-SETDB1 transformed colonies grew in medium lacking AHLT, whereas the cells co-transformed with the control GBD vector or GBD-PELP1-400 or GBD-PELP1-401-600 did not grow (Fig. 4A), suggesting a potential interaction of SETDB1 with the C-terminal region of PELP1. To verify that the observed interaction between PELP1 and SETDB1 in the yeast screen also occurs in BC cells, we performed immunoprecipitation assays using two widely used ER^+^ BC cells MCF7 and ZR75. In both models, immunoprecipitation of PELP1 followed by Western blotting confirmed that PELP1 interacts with SETDB1 (Fig. 4B). We next confirmed these interactions using SETDB1 immunoprecipitation followed by PELP1 Western blotting (Fig. 4C). Further, we validated the potential interaction between PELP1 and SETDB1 proteins using GFP-tagged PELP1 expressing MCF7 and ZR75 cell lines. In both models, GFP pull-down assays confirmed the interaction of SETDB1 with GFP-PELP1 (Fig. 4D). Since, SETDB1 knockdown decreased Akt phosphorylation in PELP1cyto cells, we examined whether cytoplasmic PELP1 interact with SETDB1 using epitope tag PELP1cyto expressing MCF7 and ZR75 cells. Immunoprecipitation with T7 tag (MCF7-PELP1cyto) and mCherry tag (ZR75-PELP1cyto) antibodies revealed that PELP1cyto interacts with endogenous SETDB1 (Fig. 4E, F). We also utilized full length GST tagged PELP1 protein to confirm PELP1 interaction with SETDB1. We incubated ZR75 whole cell lysates with GST-PELP1 and confirmed GST-PELP1 full length interacts with SETDB1 (Fig. 4G). Collectively, these results from multiple assays confirm that PELP1 is novel interacting protein of SETDB1.

### PELP1 knockdown reduces SETDB1 mediated clonogenic potential and therapy resistance

**Fig. 5:**
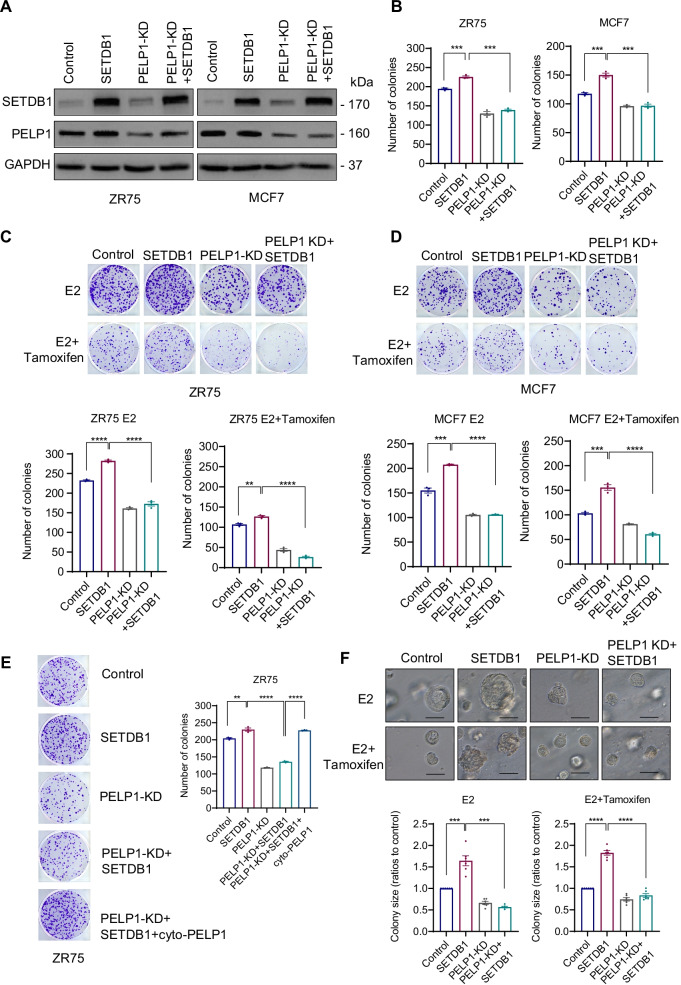
PELP1 knockdown reduces SETDB1 mediated clonogenic potential and therapy resistance. **A** Validation of ZR75 and MCF7 model cells expressing SETDB1 with/without PELP1-KD by Western blotting. **B** Characterization of the effect of PELP1-KD in cells expressing SETDB1 was analyzed by colony formation assays in 10% serum conditions. Quantitation of colonies is presented. **C**, **D** ZR75 and MCF7 model cells stably expressing with indicated constructs were cultured in E2 deprived 5% DCC medium, treated with E2 (10^–8^ M) or E2 with tamoxifen (2.5 μM) for 5 days and then cells were cultured with regular growth medium for 7 subsequent days. The image and number of colonies for each group was presented. **E** ZR75 model cells stably expressing SETDB1, PELP1 shRNA or PELP1cyto were cultured in 10% serum. The ability of PELP1cyto to rescue resistance phenotype was analyzed by colony formation assay. **F** MCF7 cells expressing SETDB1 or PELP1 shRNA were grown in 3D culture condition for 14 days in the presence of E2 (10^–8^ M) with or without tamoxifen (10 μM). Representative pictures were presented. Colony sizes were measured with ImageJ software and analyzed using relative ratio to control cells. Scale bar represents 100 μm, *n* = 6. Data was represented as mean ± SEM. **p < 0.01; ***p < 0.001; ****p < 0.0001

To further explore the functional relationship between PELP1 and SETDB1 interactions in ER^+^ BC, we established ER^+^ BC models (MCF7 and ZR75) that stably overexpress SETDB1 with and without PELP1 KD (Fig. 5A). As expected from previous studies, overexpression of SETDB1 significantly enhanced the colony formation ability, while PELP1-KD significantly attenuated the SETDB1 mediated increase in the clonogenicity of both MCF7 and ZR75 models (Fig. 5B). Further, SETDB1 overexpression models exhibited resistance to tamoxifen treatment which was decreased significantly by PELP1-KD (Fig. 5C, D). To examine whether PELP1-SETDB1 interactions in the cytoplasm contribute to the resistance phenotype, we attempted to rescue the resistance phenotype in PELP1-KD + SETDB1 clones by expressing shRNA resistant PELP1cyto plasmid. The results showed overexpression of the PELP1cyto plasmid in PELP1-KD + SETDB1 clones rescued the resistance phonotype (Fig. 5E). These results suggest that SETDB1 and PELP1 interactions in the cytoplasm are essential in promoting resistance to endocrine therapy. We then validated the effect of PELP1-KD on SETDB1 mediated oncogenic function under 3D culture conditions. Results showed that SETDB1 promoted cell growth and PELP1-KD attenuated SETDB1 driven 3D growth of BC cells (Fig. 5F). Collectively, these results support that PELP1 is needed for SETDB1 mediated growth and therapy resistance of BC cells.

### PELP1 interactions play an essential role in SETDB1 mediated Akt methylation

**Fig. 6:**
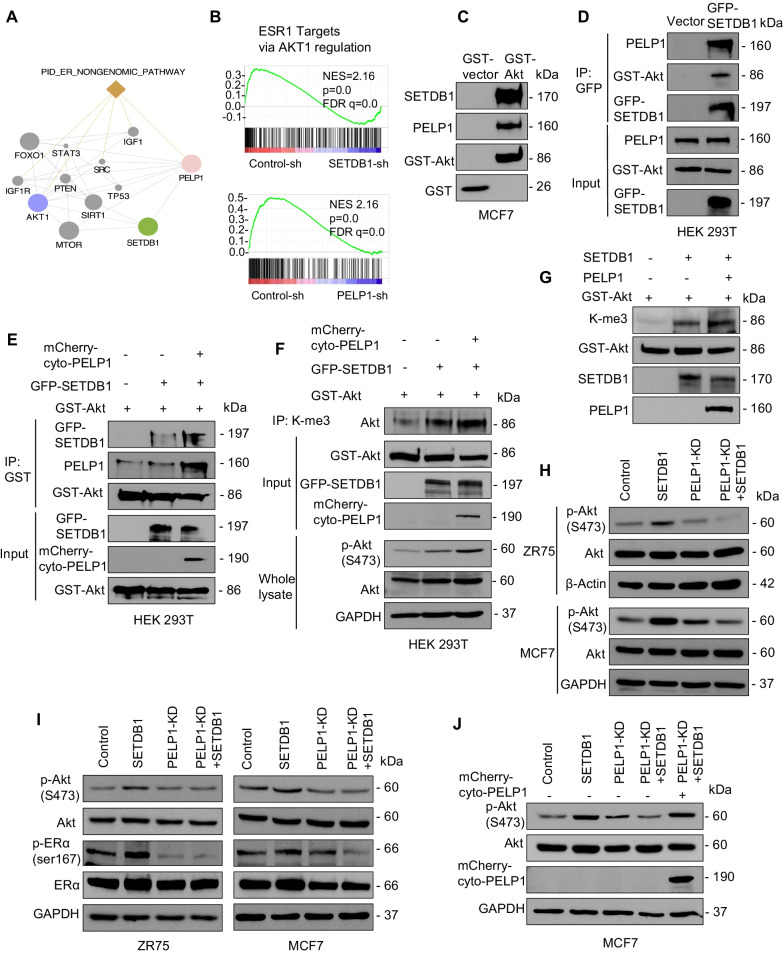
PELP1 interactions play an essential role in SETDB1 mediated Akt methylation. **A** Interactome network of PELP1, SETDB1, AKT1, and related genes based on GeneMANIA database. Each node represents genes and edges represent possible interactions. **B** GSEA plots of ESR1 targets via AKT1 regulation signature in RNA-seq data of ER^+^ BC with PELP1 or SETDB1- KD. **C** Total lysates from MCF7 cells grown in 10% serum were subjected to GST pull-down assays using GST-vector or GST-Akt purified from HEK293T cells and its ability to form trimeric complex with SETDB1, PELP1 was analyzed by Western blotting. **D** HEK293T cells are transfected with vector or SETDB1-GFP. Cells were serum-starved and stimulated with serum for 15 min and total lysates were subjected to immunoprecipitation and formation of trimeric complex was analyzed by Western blotting. **E** HEK293T cells transfected with indicated constructs were serum-starved and stimulated with serum for 15 min before being subjected to IP analysis.Western blotting was used to confirm trimeric complex. **F** Total lysates from HEK293T cells transfected with indicated constructs was subjected to immunoprecipitation using tri-methylation K-me3 antibody. Cells were serum-starved for 24 h and stimulated with 10% serum for 15 min before being subjected to IP analysis. The level of methylated Akt in immunoprecipitate and Akt phosphorylation in total cell lysate was verified by Western blotting. **G** In vitro methylation assay was performed using GST-Akt protein derived from HEK293T cells as substrate and recombinant SETDB1 protein as the source of methyltransferase. Effect of purified full-length PELP1 derived from baculovirus on SETDB1 mediated Akt methylation was determined. **H**–**J** MCF7 and ZR75 cells stably transfected with indicated constructs were serum starved for 24 h or E2 deprived for 48 h, and then stimulated with E2 (10^–8^ M, **H**) or 10% serum (**I**, **J**) for 15 min. Effect of PELP1 on SETDB1 induced Akt and ER phosphorylation was measured using Western blotting

Previous studies have shown that SETDB1 regulates Akt activation via methylation [13, 40]. Further, cytoplasmic PELP1 is also implicated in the activation of Akt leading to endocrine therapy resistance [22]. Since PELP1 interacted with SETDB1, we examined whether PELP1 plays a role in SETDB1 mediated Akt activation. Protein interaction network analyses with the GeneMANIA database predicted that SETDB1 might have physical or functional relation with PELP1 and Akt1, which are involved in ER non-genomic pathway (Fig. 6A). By comparing with previous PELP1-KD RNA-Seq enrichment data using an ER^+^ BC model, we found that both SETDB1 and PELP1 mediated genes were positively correlated with ESR1 signatures regulated via Akt (Fig. 6B). We then examined whether PELP1, SETDB1 and Akt form a trimeric complex by GST pull- down assays. MCF7 cell lysates were incubated with GST-Akt and GST pull-down assays confirmed the presence of PELP1 and SETDB1 in the eluates (Fig. 6C). We independently confirmed trimeric interaction using HEK293T cells that express GFP-tagged SETDB1. GFP pull down assays using GFP-TRAP beads confirmed trimeric interaction of Akt, SETDB1 and PELP1 proteins (Fig. 6D).

Since cytoplasmic PELP1 is implicated in the activation of Akt leading to endocrine resistance, we examined whether PELP1cyto also forms the trimeric complex using co-transfection of mCherry-PELP1cyto, or GFP-SETDB1 plasmids alone or together in GST-Akt expressing HEK293T cells. GST pull-down assays confirmed formation of the Akt trimeric complex (Fig. 6E). PELP1 is shown to modulate enzymatic activities of several methylase or demethylase enzymes by direct interactions [24, 41]. Therefore, we examined whether PELP1 affects methyltransferase activity of SETDB1 We used total lysates from model cells expressing GST-Akt, SETDB1 or PELP1 alone or together and conducted immunoprecipitation using a tri-methyl lysine (K-me3) antibody followed by Western blotting. Results showed that overexpression of PELP1cyto increased both methylation and phosphorylation of Akt mediated by SETDB1 (Fig. 6F). We then examined if PELP1 affects the methyltransferase activity of SETDB1 using an in vitro methylation assay. The results showed that addition of purified PELP1 protein enhanced SETDB1 methyltransferase activity shown by an increase in methylation of Akt (Fig. 6G).

Methylation of Akt is critical for its membrane translocation and activation. Since we confirmed that PELP1 interacts with SETDB1 and promotes its methyltransferase activity, we tested whether PELP1 is needed for SETDB1 mediated Akt activation. We generated MCF7 and ZR75 model cells stably expressing SETDB1 with or without PELP1-KD. We confirmed SETDB1 regulation of estrogen (E2) mediated Akt activation by stimulating hormone-deprived cells with E2 for 15 min. Results showed a substantial activation of Akt in SETDB1 overexpressing cells which was abolished with PELP1 KD (Fig. 6H). To confirm PELP1 function in serum-induced Akt activation, model cells were serum starved for 24 h and then stimulated with serum for 15 min. Further analyses showed increased Akt activation and ER phosphorylation at the known Akt phosphorylation site Ser167 in SETDB1 overexpressing cells which was attenuated with PELP1 KD (Fig. 6I). Finally, overexpression of shRNA resistant PELP1cyto resulted in rescuing the Akt activation in PELP1-KD + SETDB1 cells (Fig. 6J). Collectively, these results suggest that PELP1 can enhance SETDB1 mediated Akt methylation and therefore PELP1 is required for optimal activation of Akt by SETDB1.

### PELP1 is needed for SETDB1 driven ER^+^ BC progression

**Fig. 7:**
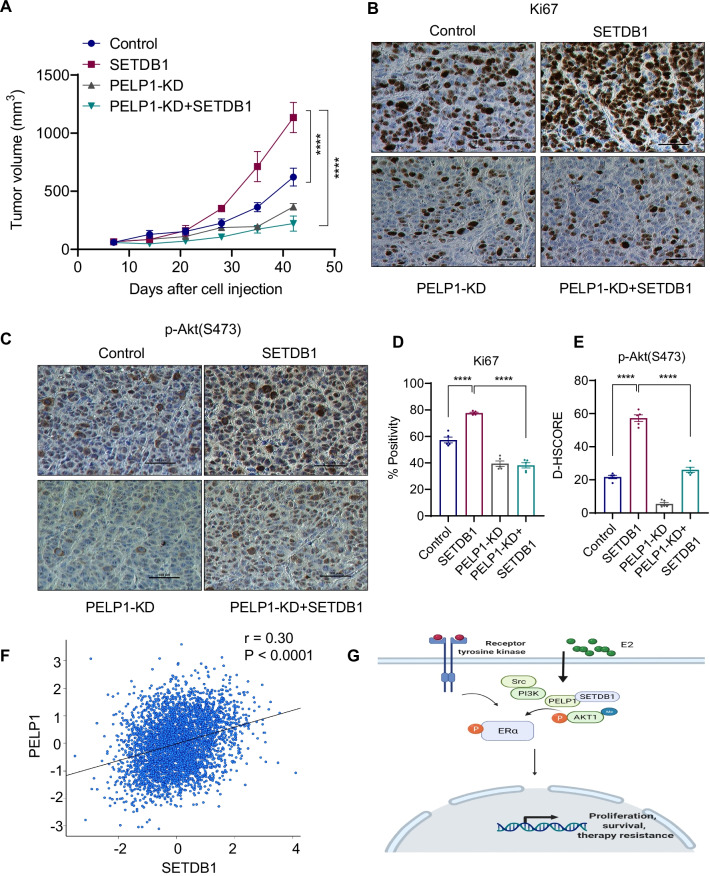
PELP1 is needed for SETDB1 mediated BC progression. **A** MCF7-Control, MCF7-SETDB1, MCF7-PELP1-KD, MCF7-PELP1-KD + SETDB1 cells were implanted into SCID mice (*n* = 5) and tumor growth was measured at indicated time points. **B–E** Tumor sections were immunostained for status of Ki-67 (**B**) and p-Akt(S473) (**C**) expression and quantitation (**D**, **E**). Representative IHC images of Ki67 and p-Akt(S473) are shown. **F** Pearson’s pairwise correlation between SETDB1 and PELP1 in ER^+^ BC patients was plotted with bc-GenExMiner using a TCGA dataset. **G** Schematic model depicting PELP1-SETDB1 axis in mediating AKT signaling in ER^+^ BC. Data was represented as mean ± SEM. ****p < 0.0001

We examined whether PELP1 is required for SETDB1 mediated ER^+^ BC progression in vivo using xenografts of MCF7 model cells that express SETDB1 with or without PELP1 KD. MCF7-SETDB1 xenografts showed increased tumor volume compared to the control vector transfected group (Fig. 7A). Interestingly, knockdown of PELP1 in SETDB1 overexpression (MCF7-PELP1KD + SETDB1) xenografts resulted in significantly reduced tumor growth compared to MCF7-SETDB1 xenografts, suggesting that PELP1 KD effectively reduces SETDB1 mediated tumor progression (Fig. 7A). Ki67 staining of the tumor sections revealed greater proliferation in the MCF7-SETDB1 xenografts compared to the MCF7-control and MCF7-PELP1 KD + SETDB1 tumors (Fig. 7B, D). Importantly, MCF7-PELP1 KD + SETDB1 tumors showed decreased expression of phospho-Akt compared to MCF7-SETDB1 tumors (Fig. 7C, E). These results suggest that PELP1 is essential for SETDB1 driven tumor growth in vivo. We also examined the human TCGA database to determine if there is a correlation between PELP1 and SETDB1 expression in ER + BC. The results showed positive correlation of PELP1 and SETDB1 in ER + BC (Fig. 7F). Collectively, these results suggest the interactions between PELP1 and SETDB1 and conclude that they play an important role in ER + BC progression by enhancing Akt signaling (Fig. 7G).

## Discussion

SETDB1 plays an oncogenic role in the progression of many cancers including breast cancer [10]. However, the mechanisms by which SETDB1 promotes endocrine therapy resistance in ER^+^ BC remain elusive. In this study, using ER + BC models we provided evidence that SETDB1 does play an important role in the regulation of subsets of ER and Akt target genes. Our studies discovered that PELP1 is a novel interacting protein of SETDB1 and demonstrated PELP1 is needed for SETDB1 mediated oncogenic functions and endocrine therapy resistance. Further, our studies showed that PELP1 plays an essential role in the activation of oncogenic Akt signaling by enhancing SETDB1 mediated methylation of Akt. Finally, we demonstrated that PELP1 plays a critical role in SETDB1 mediated tumor progression in vivo. Collectively, these findings implicate the important role of the PELP1/SETDB1 axis in ER^+^ BC progression and development of endocrine therapy resistance.

SETDB1 is a known oncogene, and its high expression correlate with worse overall survival, and shorter relapse-free survival [42]. SETDB1 enhances c-MYC and cyclin D1 expression, and thus provides a growth advantage to breast cancer cells [43]. In addition, SETDB1 is also known to regulate metastases in TNBC cells [44] and EMT by regulating expression of the transcription factor Snail [45]. Our results using RNA-Seq showed that SETDB1-regulated genes are positively correlated with ER signaling, tamoxifen resistance and PI3K-Akt signaling. Our studies identified PELP1 as a novel interactor of SETDB1 using yeast based two hybrid screen and PELP1 enhances SETDB1 mediated Akt methylation. Further, SETDB1 and PELP1-regulated genes in ER^+^ BC are positively correlated with ESR1 targets via Akt1 signature. RNA-Seq data suggested SETDB1 regulate a number of pathways in BC cells and some of these pathways are common to PELP1 regulated pathways, while others are PELP1 independent. Our previous studies showed that cytoplasmic PELP1 signaling contributes to endocrine resistance; however the mechanism is not clear. In the present study, our results suggest that PELP1/SETDB1 complex mediated extranuclear functions and endocrine resistance occur via AKT1 and PELP1 knockdown compromises these functions. However, in this study, we did not examine the other SETDB1 mediated functions including epigenetic changes, p53 signaling, cell cycle, EMT, and metastases. Future studies are needed to determine whether PELP1plays a role in other SETDB1 mediated functions.

SETDB1 is a methyltransferase involved in di and tri-methylation of histone H3 at K9 [9] Recent studies identified that SETDB1 also methylates non-histone substrates. In fact, SETDB1 interacts with Akt [46], methylates Akt at K64 or K140 to elicit Akt ubiquitination, cell membrane recruitment, phosphorylation and subsequent activation upon stimulation by growth factors [13, 40]. Our results also suggest that PELP1 plays an essential role in the activation of Akt by SETDB1. Using in vitro and in vivo methylation assays, we provided evidence that PELP1 enhances the methylation of Akt by SETDB1. Furthermore, knockdown of PELP1 substantially reduced SETDB1 mediated Akt methylation, Akt phosphorylation and ER phosphorylation. A limitation of mechanistic studies and biological assays conducted in our study is that it only focused on SETDB1-mediated extra nuclear functions via Akt1 that contribute to endocrine resistance and cell survival. Since both PELP1 and SETDB1 are also present in the nucleus, they may have additional functions in the nuclear compartment. Future studies are needed to examine the significance of PELP1 on SETDB1 mediated nuclear functions.

PELP1 oncogenic signaling is implicated in the progression of several cancers including breast r [47]. It is well documented that PELP1 expression is an independent prognostic predictor of shorter breast cancer–specific survival and disease-free interval [18], and PELP1 dysregulation contributes to BC therapy resistance [19, 20]. Hormonal therapies using tamoxifen and fulvestrant induce a pro-invasive and pro-migratory phenotype in ER^+^ BC and exhibit a high basal expression of PELP1 [48]. The PELP1/SRC-3-dependent regulation of metabolic PFKFB kinases has been shown to drive therapy resistant ER + BC [49] and cytoplasmic PELP1/SRC-3 signaling complexes increase BC stem cells [50]. ER + BC tumors with high levels of cytoplasmic PELP1 exhibit poor response to tamoxifen treatment [21]. Our data suggests that SETDB1-PELP1 interactions play a key role in endocrine resistance and that the PELP1 localization to the cytoplasm, commonly seen in breast tumors, may play a role in enhancing SETDB1 oncogenic signaling. Further, our results suggest that PELP1 plays an essential role in SETDB1 mediated endocrine therapy resistance as PELP1 knockdown reduced SETDB1 mediated therapy resistance to endocrine therapies.

Our analyses of TCGA datasets revealed that both SETDB1 and PELP1 are commonly overexpressed in BC. We found that endocrine therapy resistant models express higher levels of SETDB1 and the down regulation of SETDB1 expression sensitizes them to endocrine therapy. Our results also suggested that PELP1 plays an essential role in SETDB1 mediated BC progression to endocrine therapy resistance. Further, our results support that SETDB1 is a novel PELP1 binding protein. Accordingly, SETDB1 expression is positively correlated with PELP1 expression in ER+ BC patients. Since, dysregulated Akt signaling has been linked to ER + BC progression and endocrine therapy resistance, we predict that the commonly deregulated SETDB1-PELP1 axis may contribute to hyperactive Akt signaling leading to BC progression and endocrine therapy resistance. As of now, no specific inhibitors of SETDB1 are currently available. Previous studies have demonstrated that Mithramycin A reduce the expression of SETDB1 by targeting the SP1 transcription factor which regulate expression of SETDB1 [51, 39]. Our results using SETDB1 KD or reduction of SETDB1 levels via Mithramycin A treatment provided evidence for the utility of targeting SETDB1 in sensitizing endocrine therapy resistant ER^+^ BC cells. However, development of a specific inhibitor of SETDB1 is needed for future translation of these findings.

## Conclusions

In summary, our data provides the first evidence demonstrating PELP1 as a novel interacting protein of SETDB1 and that PELP1 is essential for optimal SETDB1 mediated Akt activation. Further, these results suggest that PELP1/SETDB1 interactions play an important role in Akt activation and in the development of endocrine therapy resistance. Drugs that target the PELP1/SETDB1 axis may be useful in blocking aberrant Akt signaling in ER^+^ and therapy resistant BC.

## Supplementary Information

**Additional file 1**. List of primer sequences.

## Data Availability

All data generated for this study are included within this article and in the supplementary information. RNA-seq data has been deposited in the GEO database under a GEO accession number GSE187398.

## Abbreviations

ER: Estrogen receptor
BC: Breast cancer
ER^+^: Estrogen receptor alpha positive
SETDB1: SET domain bifurcated 1
PELP1: Proline-, glutamic acid-, and leucine-rich protein 1
TNBC: Triple negative breast cancers
EMT: Epithelial-mesenchymal transition
ATCC: American-Type Culture Collection
MCF7-TamR: Tamoxifen-resistant MCF7 cells
MCF7-FR: Fulvestrant-resistant MCF7 cells
E2: Estrogen
GAD: Gal4 activation domain
GBD: Gal4 binding domain
LT: Leucine and tryptophan
AHLT: Adenine, histidine, leucine, and tryptophan
SAM: S-adenosyl-methionine
GSEA: Gene set enrichment analysis
IHC: Immunohistochemistry
KD: Knock down
PELP1cyto: PELP1 cytoplasmic localization
K-me3: Tri-methyl lysine
