# Supplementary material for: SETDB1 interactions with PELP1 contributes to breast cancer endocrine therapy resistance
Source: Breast Cancer Res. 2022 Apr 8;24:26. doi: 10.1186/s13058-022-01520-4 (PMC8991965; doi:10.1186/s13058-022-01520-4)
Supplement: Supplementary file 1 — Additional file 1. List of primer sequences. [file 13058_2022_1520_MOESM1_ESM.docx]

| List of primers used in this study | | |
| --- | --- | --- |
| **Gene name** | **Forward Primer** | **Reverse Primer** |
| *FOXC1* | TGTTCGAGTCACAGAGGATCG | ACAGTCGTAGACGAAAGCTCC |
| *PGR* | ACCCGCCCTATCTCAACTACC | AGGACACCATAATGACAGCCT |
| *S100A9* | GGTCATAGAACACATCATGGAGG | GGCCTGGCTTATGGTGGTG |
| *SERPINA1* | GGAGGCTCAGATCCATGAAGG | GGTGTCCCCGAAGTTGACAG |
| *CCNA1* | ACATGGATGAACTAGAGCAGGG | GAGTGTGCCGGTGTCTACTT |
| *EGR3* | GACATCGGTCTGACCAACGAG | GGCGAACTTTCCCAAGTAGGT |
| *IGF1R* | TCGACATCCGCAACGACTATC | CCAGGGCGTAGTTGTAGAAGAG |
| *PDZK1* | GAACTGCCCAATGGCTCTGT | CCCCGAATCGCATTTAAGTGAA |
| *FGFBP2* | TTCCTGCACTATGCGTCCC | GGGCTTGATTCCAGTAAGGTTT |
| *TFF1* | TTGTGGTTTTCCTGGTGTCA | CCGAGCTCTGGGACTAATCA |
| *MYC* | CCTACCCTCTCAACGACAGC | CTCTGACCTTTTGCCAGGAG |
| *E2F1* | CGCATCTATGACATCACCAACG | GAAAGTTCTCCGAAGAGTCCACG |
| *GREB1* | GGCAGGACCAGCTTCTGA | CTGTTCCCACCACCTTGG |
| *RET* | ACACGGCTGCATGAGAACAA | GCCCTCACGAAGGGATGTG |
| *TFAP2C* | TCAGTCCCTGGAAGATTGTCG | CCAGTAACGAGGCATTTAAGCA |
| *GAPDH* | GGAGCGAGATCCCTCCAAAAT | GGCTGTTGTCATACTTCTCATGG |
